# A hippocampal circuit linking dorsal CA2 to ventral CA1 critical for social memory dynamics

**DOI:** 10.1038/s41467-018-06501-w

**Published:** 2018-10-09

**Authors:** Torcato Meira, Felix Leroy, Eric W. Buss, Azahara Oliva, Jung Park, Steven A. Siegelbaum

**Affiliations:** 1 Department of Neuroscience, Zuckerman and Kavli Institutes, Vagelos College of Physicians and Surgeons, Columbia University, 3227 Broadway, New York, NY 10027 USA; 2 Life and Health Sciences Research Institute (ICVS), School of Medicine, University of Minho, Braga, 4710-057 Portugal; 3 ICVS/3B’s - PT Government Associate Laboratory, Braga/Guimarães, 4806-909 Portugal

## Abstract

Recent results suggest that social memory requires the dorsal hippocampal CA2 region as well as a subset of ventral CA1 neurons. However, it is unclear whether dorsal CA2 and ventral CA1 represent parallel or sequential circuits. Moreover, because evidence implicating CA2 in social memory comes largely from long-term inactivation experiments, the dynamic role of CA2 in social memory remains unclear. Here, we use pharmacogenetics and optogenetics in mice to acutely and reversibly silence dorsal CA2 and its projections to ventral hippocampus. We show that dorsal CA2 activity is critical for encoding, consolidation, and recall phases of social memory. Moreover, dorsal CA2 contributes to social memory by providing strong excitatory input to the same subregion of ventral CA1 that contains the subset of neurons implicated in social memory. Thus, our studies provide new insights into a dorsal CA2 to ventral CA1 circuit whose dynamic activity is necessary for social memory.

## Abstract (web summary)

Although the CA2 region of the hippocampus has been implicated in social memory, its precise role has been unclear. Here, the authors show that the dorsal subregion of CA2 is required for the encoding, consolidation and recall of social memory through a circuit linking it to ventral CA1.

## Introduction

The hippocampus (HPC) is an extensively studied structure with a well-defined role in declarative memory, which includes memory of places, people, events, and facts^1^. As declarative memory consists of distinct stages, including encoding, consolidation, and recall, a key question is the extent to which different hippocampal subregions participate in distinct aspects of declarative memory. Indeed, some studies have revealed specific roles of several hippocampal subregions in memory, including the importance of dentate gyrus (DG) for pattern separation^2^, CA3 for pattern completion and one-trial contextual learning^3^, and direct cortical inputs to CA1/subiculum for temporal association memory^4^. However, to date, relatively little is known about the role of CA2 in declarative memory, even though it has distinct synaptic connectivity and morphological, electrophysiological, and molecular characteristics^5^.

An important role for dorsal CA2 (dCA2) in social memory was demonstrated using a genetically engineered mouse line (*Amigo2*-Cre) that allowed for the selective silencing of dCA2 pyramidal neurons (PNs) by expression of tetanus neurotoxin (TeNT) light chain^6^. Importantly, dCA2 PNs silencing did not have any impact on sociability or several HPC-dependent behaviors, including spatial memory, contextual memory, and novel object recognition. Excitotoxic lesion of dCA2 was also found to impair social memory^7^. More recently, Smith et al.^8^ demonstrated that stimulation of vasopressinergic axons from the paraventricular nucleus in area dCA2 during social memory encoding enhances social recognition memory through activation of the CA2-enriched arginine vasopressin 1b receptor (AVPR1b).

However, approaches used to inactivate dCA2 up to now have resulted in irreversible silencing of dCA2^6,7^, which may produce consequent long-term reactive changes in brain regions outside of dCA2 that may contribute to the socio-cognitive deficits. Indeed, chronic inactivation of dCA2 PNs transmission with TeNT was recently shown to perturb the hippocampal dynamic excitatory/inhibitory balance^9^. Moreover, long-term silencing studies cannot determine whether dCA2 is necessary for encoding, consolidation or recall of social memory.

In addition to dCA2, ventral CA1 (vCA1)^10^ and ventral CA3 (vCA3)^11^ have also been implicated in social memory. Moreover, Okuyama et al.^10^ found that it is the subset of vCA1 PNs that project to the nucleus accumbens (NAc) shell that are important for social memory. However, it is unknown whether dCA2 and vCA1/vCA3 hippocampal regions are integrated into a single social memory circuit or whether they provide parallel circuits necessary for social memory.

Hence, to explore these questions, we used pharmacogenetic and optogenetic approaches to acutely and reversibly inactivate dCA2 and its projections to ventral HPC (vHPC)^10,12^ during different phases of social memory tasks. We find that dCA2 plays an active role in social memory encoding, consolidation, and recall. Furthermore, we show that dCA2 participates in social memory by providing an excitatory input to the same vCA1 subregion that projects to the NAc shell.

## Results

### Acute dCA2 silencing impairs social memory

**Fig. 1:**
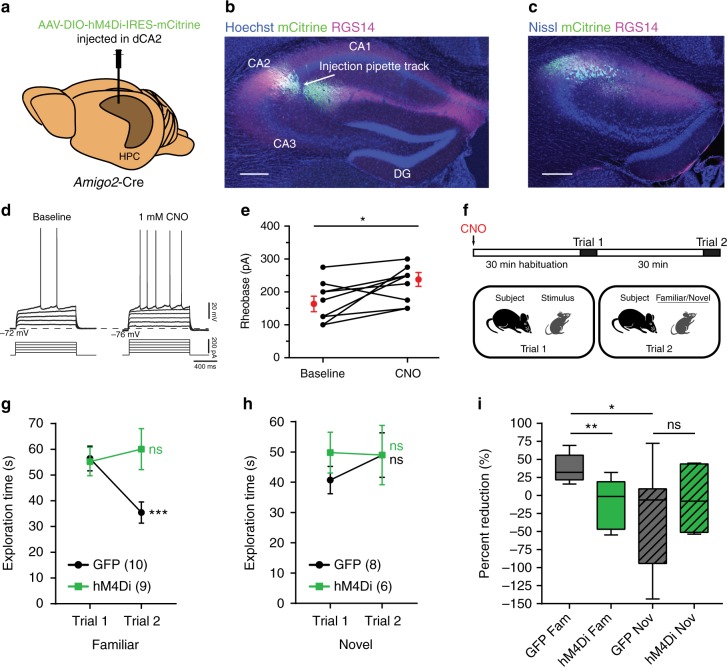
Acute dCA2 silencing impairs social memory. **a** AAV-DIO-hM4Di-IRES-mCitrine injection into dCA2 of *Amigo2*-Cre mice. **b** Overlap of mCitrine (green) and RGS14 (magenta). **c** Anterior extent of mCitrine expression. **d** Action potentials recorded from hM4Di-expressing CA2 PNs (*n* = 10) in response to progressively higher depolarizing currents before (baseline) and after CNO application. **e** Rheobase is increased after CNO application (paired *t* test: *t*(9) = 2.967, *P* = 0.0158). **f** Direct interaction test of social memory performed 30 min after CNO IP in hM4Di-expressing and control (GFP-expressing) *Amigo2*-Cre mice. **g** When the same stimulus mouse encountered in trial 1 is also encountered in trial 2, GFP-expressing but not hM4Di-expressing mice showed decreased social exploration time (two-way ANOVA: treatment × trial *F*(1,17) = 14.85, *P* = 0.0013; GFP: *n* = 10, *P* = 0.0006, Sidak’s multiple comparisons test; hM4Di: *n* = 9, *P* = 0.5572, Sidak’s multiple comparisons test). **h** When a novel stimulus mouse was encountered in trial 2, neither GFP-expressing nor hM4Di-expressing mice showed decreased social exploration (two-way ANOVA: treatment × trial *F*(1,12) = 0.5303, *P* = 0.4805; GFP: *n* = 8, *t*(7) = 1.078, *P* = 0.3169, paired *t* test; hM4Di: *n* *=* 6, *t*(5) = 0.07924, *P* = 0.9399, paired *t* test). **i** Reduction in social exploration time in trial 2 by groups from **g** and **h**. For GFP-expressing mice, reduction in exploration during trial 2 to the same (Fam, familiar) mouse presented in trial 1 was significantly greater than reduction when a novel (Nov) mouse was presented in trial 2 (unpaired *t* test: *t*(16) = 2.851, *P* = 0.0116) or when hM4Di-expressing mice encountered a familiar mouse in trial 2 (unpaired *t* test: *t*(17) = 3.962, *P* = 0.0010). No difference was found between GFP and hM4Di-expressing mice encountering a novel mouse in trial 2 (unpaired *t* test: *t*(12) = 0.6749, *P* = 0.5126). Results in **e**, **g** and **h** show mean ± s.e.m. Box-whiskers plot in **i** present median (center line), 25th to 75th percentiles (box) and minimal and maximal values (whiskers). *P < 0.05; **P < 0.01; ***P < 0.001; ns, *P* > 0.05. Scale bars, 250 µm (**b** and **c**)

To evaluate the impact of acute dCA2 activity in social memory, we expressed the inhibitory DREADD (Designer Receptor Exclusively Activated by Designer Drug) hM4Di receptor^13^ selectively in dCA2 PNs by injecting Cre-dependent rAAV2/2 hSyn.DIO.HA-hM4D(Gi).IRES.mCitrine (which we refer to as AAV-DIO-hM4Di-IRES-mCitrine) into the dCA2 hippocampal region of *Amigo2*-Cre mice (Fig. 1a). Cre expression is largely restricted to CA2 PNs in this line in mice 5–6 weeks of age or older^6^. We first confirmed the neuronal specificity of viral expression by imaging mCitrine, whose expression was confined to neurons co-expressing the CA2-specific marker RGS14 (Fig. 1b) (99.41 ± 0.59% overlap; mean ± standard error of mean—s.e.m.). Our injections resulted in viral-mediated expression in the most anterior third of dCA2 (Fig. 1c), with about half of all RGS14-positive neurons at the site of injection expressing mCitrine (48.07 ± 6.87% cells, *n* = 13 mice).

We previously verified the functional efficacy of this approach by showing that the hM4Di agonist clozapine N-oxide (CNO) produced a reversible 8 mV hyperpolarization of CA2 PNs expressing hM4Di in hippocampal slices^14^. CNO also reduced the excitability of hM4Di-expressing CA2 PNs, as shown by an increase in the minimal depolarizing current required to elicit an action potential (rheobase) (Fig. 1d, e).

Next, we examined the impact of acute dCA2 silencing on social memory. Because CNO or a metabolite may exert off-target actions, we compared the effect of intraperitoneal (IP) injection of CNO in *Amigo2*-Cre mice infected in dCA2 with either AAV-DIO-hM4Di-IRES-mCitrine or a GFP-expressing AAV. Three weeks after viral infection, both groups were injected with CNO IP (10 mg kg^−1^) and then tested 30 min later in a direct interaction social memory task. In this task, a subject mouse was exposed to a novel juvenile stimulus mouse for 2 min on trial 1 (encoding trial). After a 30 min intertrial interval, the subject mouse was re-exposed to either the same (now familiar) stimulus mouse for 2 min on trial 2 (recall trial) or to a second novel juvenile stimulus mouse (Fig. 1f). Social memory is expressed as a decrease in the time of exploration of the same stimulus mouse from trial 1 to trial 2, as seen in the GFP-expressing mice. In contrast, the hM4Di-expressing mice failed to show a decrease (Fig. 1g, i), indicative of impaired social memory. The decrease in social exploration of the stimulus mouse displayed by the control GFP-expressing mice did not result from fatigue or disengagement on trial 2 as neither GFP- nor hM4Di-expressing mice showed a significant decrease in exploration time when a novel juvenile was encountered on trial 2 (Fig. 1h, i).

In principle, impairments in social memory behavior can result from alterations in locomotion or sociability (the normal behavior of an animal to engage in social exploration). However, the duration of social exploration during trial 1 was similar in both groups (unpaired *t* test: *t*(17) = 0.1694, *P* = 0.8675), suggestive of intact sociability. Additionally, we performed a specific test of sociability using a three-chamber apparatus, in which a subject mouse is allowed to explore a chamber containing a novel mouse inside a wire cup versus a chamber with an empty wire cup (Supplementary Fig. 1a). CNO injection into *Amigo2*-Cre-positive and *Amigo2*-Cre-negative (WT) mice previously injected in dCA2 with AAV-DIO-hM4Di-IRES-mCitrine had no effect on sociability as both groups similarly preferred the chamber containing the novel mouse (Supplementary Fig. 1b, c). Silencing dCA2 also had no effect on locomotion in two different tasks (Supplementary Fig. 2).

From these experiments we conclude that acute activity of dCA2 PNs is necessary for social memory but not for sociability. Next, we take advantage of the ability of pharmacogenetic and optogenetic approaches to acutely and reversibly silence dCA2 to determine whether dCA2 is important for social memory encoding, consolidation and/or recall.

### dCA2 is necessary in social memory formation

**Fig. 2:**
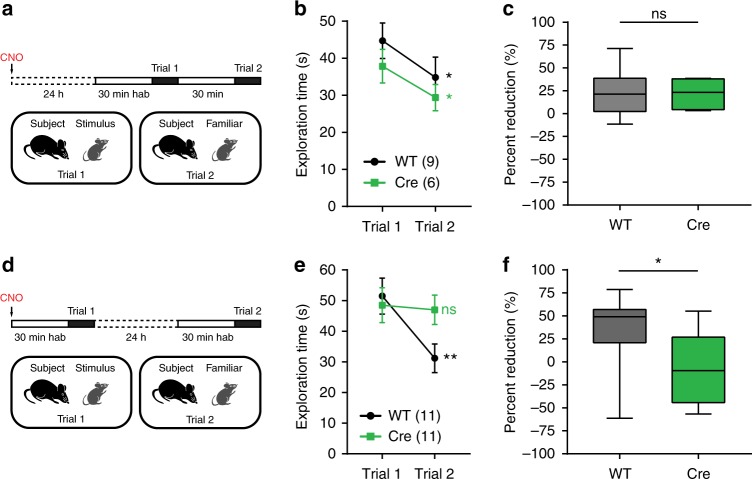
dCA2 is necessary in the initial stages of social memory formation. **a** Protocol for experiment examining effect of IP injection of CNO 24 h prior to the direct interaction test. Both WT and *Amigo2*-Cre littermates were injected in dCA2 with AAV-DIO-hM4Di-IRES-mCitrine. **b** WT controls and *Amigo2*-Cre mice showed normal decrease in social exploration time during trial 2 (WT: *n* = 9, *t*(8) = 2.815, *P* = 0.0227, paired *t* test; Cre: *n* *=* 6, *t*(5) = 2.790, *P* = 0.0384, paired *t* test). The groups did not differ significantly (two-way ANOVA: treatment × trial *F*(1,13) = 0.08127, *P* = 0.7801). **c** No difference was found in percent reduction in social exploration time in trial 2 compared to trial 1 between groups (unpaired *t* test: *t*(13) = 0.1591, *P* = 0.8760). **d** Protocol for a direct interaction test with 24 h intertrial interval. WT and *Amigo2*-Cre littermates were injected in dCA2 with AAV-DIO-hM4Di-IRES-mCitrine. Trial 1 was performed 30 min after IP injection of CNO and trial 2 was performed 24 h later. **e** WT but not *Amigo2*-Cre mice displayed decreased social exploration during trial 2 relative to trial 1 (WT: *n* = 11, *P* = 0.0040, Sidak’s multiple comparisons test; Cre: *n* *=* 11, *P* = 0.9578, Sidak’s multiple comparisons test). The groups differed significantly (two-way ANOVA: treatment × trial *F*(1,20) = 5.394, *P* = 0.0309). **f** Percent reduction in social exploration time was lower in *Amigo2*-Cre than WT groups (unpaired *t* test: *t*(20) = 2.558, *P* = 0.0187). Results in **b** and **e** show mean ± s.e.m. Box-whiskers plots in **c** and **f** present median (center line), extension from the 25th to 75th percentiles (box) and minimal and maximal values (whiskers). *P < 0.05; **P < 0.01; ns, *P* > 0.05

We first examined whether dCA2 was necessary during the initial phases of social memory formation, including both encoding and consolidation, using DREADD/CNO-silencing of dCA2 in a direct interaction test with a 24 h intertrial interval. The physiological effects of IP injection of CNO mediated by DREADD activation last for several hours but are reversible within 24 h^15,16^. Thus, by applying CNO by IP injection 30 min prior to trial 1, we could silence dCA2 during memory encoding in trial 1 and for the next several hours after the animal is returned to its home cage. However, CNO should no longer be effective 24 h after its administration during memory recall in trail 2. We first confirmed that the action of CNO was indeed reversible within this time frame by injecting the compound 24 h prior to performing a direct interaction test with a 30 min intertrial interval in *Amigo2*-Cre mice or WT littermates injected previously with AAV-DIO-hM4Di-IRES-mCitrine. Under these conditions, CNO injection had no effect on social memory, in contrast to the efficacy of CNO when injected 30 min prior to trial 1 in mice expressing hM4Di (compare Fig. 2a–c to Fig. 1f, g, i).

Next, we confirmed that our grouped-housed mice displayed social memory in a direct interaction test with a 24 h interval between encoding and recall^17^. Indeed, we found that subject mice showed a significant decrease in exploration time of the familiar juvenile stimulus mouse during trial 2 compared to trial 1 (Supplementary Fig. 3a, b). This decrease represents social memory of the stimulus mouse encountered on trial 1 rather than fatigue or disengagement on trial 2 as subject mice showed an undiminished social exploration time when they were presented with a novel juvenile stimulus mouse on trial 2 (Supplementary Fig. 3a–c).

Finally, to examine the importance of dCA2 during encoding/consolidation, we performed a direct interaction test with a 24 h interval with CNO injected IP 30 min prior to trial 1 in mice that had been previously injected in dCA2 with AAV-DIO-hM4Di-IRES-mCitrine (Fig. 2d). The WT mice showed a significant decrease in social exploration from trial 1 (day 1) to trial 2 (day 2) after a 24 h interval. In contrast, the *Amigo2*-Cre mice showed no decrease in exploration time (Fig. 2e, f), indicating an impairment in social memory encoding and/or consolidation. As a further control to examine whether the deficit in social memory in *Amigo2*-Cre mice was specific for the action of CNO to activate hM4Di, and not due to the Cre transgene or hM4Di expression alone, we examined the effect of saline injected IP 30 min prior to trial 1 in *Amigo2*-Cre mice and WT littermates, both previously injected in dCA2 with AAV-DIO-hM4Di-IRES-mCitrine. Saline-injected *Amigo2*-Cre mice and WT littermates both had intact social memory 24 h after trial 1, indicating that the effect of CNO in *Amigo2*-Cre mice expressing hM4Di was indeed specific (Supplementary Fig. 4).

### dCA2 is necessary for social memory encoding

As CNO is effective for several hours after its injection, the above experiment does not reveal whether dCA2 is necessary for social memory encoding during the 2 min period of social exploration in trial 1 as opposed to memory consolidation, which occurs during a several hour period following trial 1^18,19^. Thus, to determine if dCA2 participates in social memory encoding, we used an optogenetic approach to silence dCA2 only during encoding in trial 1. We injected *Amigo2*-Cre and WT mice in the dCA2 region with Cre-dependent rAAV2/5 EF1a.DIO.eArch3.0-eYFP (AAV-DIO-eArch3.0-eYFP) to express the light-activated proton pump eArch3.0. This led to specific expression in RGS14-expressing CA2 PNs in *Amigo2*-Cre mice (96.94 ± 1.56% overlap) in a large fraction of neurons, with 84.13 ± 2.66% of RGS14-positive cells expressing eArch3.0-eYFP at the injection site (*n* = 4 mice). To determine whether eArch3.0 was able to inhibit dCA2 activity, we implanted a combined multimode optic fiber and multishank silicon probe in dCA2. Indeed, 1-s pulses of green light (5 mW at 593 nm) significantly decreased the firing rate of dCA2 PNs (Supplementary Fig. 5).

**Fig. 3:**
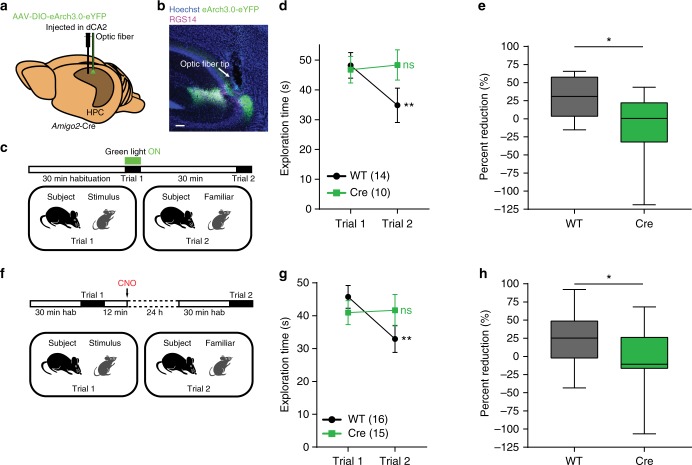
dCA2 is necessary for social memory encoding and consolidation. **a** Diagram illustrating injection of AAV-DIO-eArch3.0-eYFP into dCA2 of *Amigo2*-Cre and WT littermates. An optic fiber was implanted above dCA2. **b** Dorsal HPC coronal section from an *Amigo2*-Cre mouse expressing eArch3.0-eYFP in dCA2 (green). Arrow indicates optic fiber tip location, above dCA2. **c** Behavior protocol for testing whether dCA2 is required for social memory encoding. Green light was delivered through the optic fiber during the 2 min of trial 1 of a direct interaction test. Trial 2 was performed 30 min later with light off. **d** WT mice displayed decreased social exploration during trial 2 (WT: *n* = 14, *P* = 0.0074, Sidak’s multiple comparisons test) whereas *Amigo2*-Cre mice did not (Cre: *n* *=* 10, *P* = 0.9371, Sidak’s multiple comparisons test). The groups differed significantly (two-way ANOVA: treatment × trial *F*(1,22) = 5.482, *P* = 0.0287). **e** Percent reduction in social exploration time was lower in *Amigo2*-Cre mice than WT littermates (unpaired *t* test: *t*(22) = 2.590, *P* = 0.0167). **f** Protocol for determining whether dCA2 is needed during memory consolidation with a direct interaction test. WT and *Amigo2*-Cre littermates were injected in dCA2 with AAV-DIO-hM4Di-IRES-mCitrine. CNO was given IP 12 min after trial 1, with trial 2 performed 24 h later. **g** Only WT mice displayed social memory (WT: *n* = 16, *P* = 0.0045, Sidak’s multiple comparisons test; Cre: *n* = 15, *P* = 0.9797, Sidak’s multiple comparisons test). The groups differed significantly (two-way ANOVA: treatment × trial *F*(1,29) = 6.058, *P* = 0.020). **h** Percent reduction was lower in *Amigo2*-Cre compared to WT group (unpaired *t* test: *t*(29) = 2.173, *P* = 0.0381). Results in **d** and **g** show mean ± s.e.m. Box-whiskers plots in **e** and **h** present median (center line), extension from the 25th to 75th percentiles (box) and minimal and maximal values (whiskers). *P < 0.05; **P < 0.01; ns, *P* > 0.05. Scale bar, 250 µm (**b**)

Next, we examined the effect of optogenetic silencing of dCA2 during social memory encoding. *Amigo2*-Cre mice and WT littermates were injected in the dCA2 area with AAV-DIO-eArch3.0-eYFP and, after 1 week, mice were implanted with bilateral ferrules for optic fiber probes above dCA2 (Fig. 3a, b). Three weeks after viral injection, both groups were tested in the direct interaction social memory test with a 30 min interval between trial 1 and trial 2, with green light delivered to dCA2 during the entire 2 min period of trial 1 (Fig. 3c). WT mice displayed intact social memory as evidenced by a decreased social exploration time during trial 2. In contrast, *Amigo2*-Cre eArch3.0-expressing mice showed no significant decrease in social exploration from trial 1 to trial 2 (Fig. 3d, e). When eArch3.0-expressing *Amigo2*-Cre mice were retested in the same direct interaction test but without light delivery, they showed intact social memory (paired *t* test, *n* = 6: *t*(5) = 6.515, *P* = 0.0010), indicating the specificity of the light-evoked activation of eArch3.0.

### dCA2 is necessary for social memory consolidation

Is dCA2 also required for consolidation? We addressed this question in a direct interaction test with a 24 h intertrial interval by injecting CNO IP soon after trial 1. As CNO acts for several hours but is no longer effective 24 h after administration (Fig. 2a–c), at the time of recall in trial 2, any memory deficit must indicate a role for dCA2 during memory consolidation. *Amigo2*-Cre and WT littermates were first injected with AAV-DIO-hM4Di-IRES-mCitrine in dCA2 and then, after 3 weeks, injected with CNO IP 12 min after social memory encoding in trial 1 (Fig. 3f). WT control mice displayed a significant decrease in exploration time during trial 2. In contrast, *Amigo2*-Cre mice showed no decrease in exploration time (Fig. 3g, h), consistent with the view that dCA2 is necessary for social memory consolidation.

**Fig. 4:**
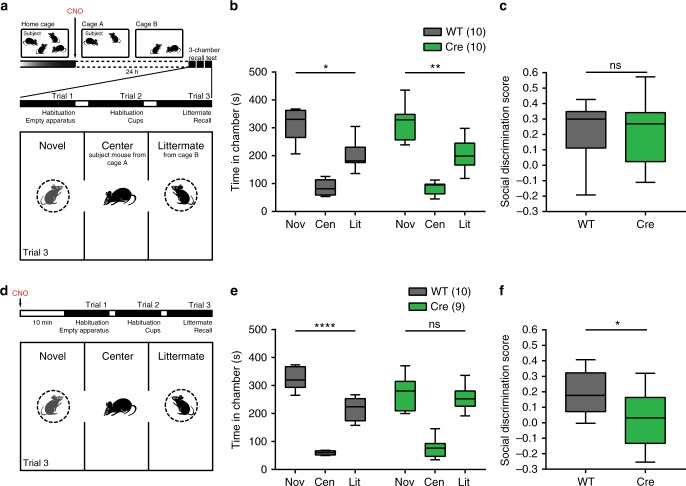
dCA2 silencing does not disrupt a well-consolidated memory of a littermate but prevents its recall. **a** Protocol testing effect of dCA2 silencing on retention of a well-consolidated social memory. WT and *Amigo2*-Cre mice were injected with AAV-DIO-hM4Di-IRES-mCitrine in dCA2. Mice were given CNO IP and randomly divided into two housing cages (A and B). After 24 h, subject mice from cage A were presented with a novel (Nov) mouse and a littermate (Lit) from cage B. **b** WT and *Amigo2*-Cre mice spent more time in the chamber housing the novel animal (WT repeated measures one-way ANOVA: *n* = 10, *F*(1.414, 12.73) = 44.89, *P* < 0.0001, Novel vs Littermate *P* = 0.0114, Tukey’s multiple comparisons test; Cre repeated measures one-way ANOVA: *n* = 12, *F*(1.253, 13.79) = 50.15, *P* < 0.0001, Novel vs Littermate *P* = 0.0086, Tukey’s multiple comparisons test). The groups did not differ significantly (two-way ANOVA: treatment × chamber *F*(2,40) = 0.006505, *P* = 0.9935). **c** WT and *Amigo2*-Cre mice showed similar social discrimination scores (unpaired *t* test: *t*(20) = 0.03401, *P* = 0.9732). **d** Experimental protocol silencing dCA2 during social memory recall. WT and *Amigo2*-Cre mice previously injected with AAV-DIO-hM4Di-IRES-mCitrine were given CNO IP 30 min prior to the social discrimination trial. **e** WT mice spent more time in the novel animal chamber compared to the littermate chamber; hM4Di-expressing *Amigo2*-Cre mice did not show a preference (WT: *n* = 10, *P* < 0.0001, Tukey’s multiple comparisons test; Cre: *n* = 9, *P* = 0.8026, Tukey’s multiple comparisons test). The groups differed significantly (two-way ANOVA: treatment × chamber *F* (2,34) = 5.005, *P* = 0.0124). **f** *Amigo2*-Cre mice social discrimination score was significantly lower than that of the WT group (unpaired *t* test *t*(17) = 2.391, *P* = 0.0286). Box-whiskers plots present median (center line), extension from the 25th to 75th percentiles (box) and minimal and maximal values (whiskers). *P < 0.05; **P < 0.01; ****P < 0.0001; ns, *P* > 0.05

An alternative explanation for the social memory deficit seen in this protocol is that some level of continuous dCA2 activity may be required to maintain a social memory even after consolidation, rather than being indicative of a need for dCA2 activity during the consolidation process itself. To test this idea, we examined whether dCA2 silencing could disrupt a long-term well-consolidated memory of a co-housed littermate. *Amigo2*-Cre mice and WT littermates, previously injected in dCA2 with AAV-DIO-hM4Di-IRES-mCitrine, were given CNO IP and randomly divided into two cages (A and B) where they were housed for 24 h. If continuous dCA2 activity were required to maintain a consolidated social memory, subject mice from cage A in which dCA2 was silenced should no longer recognize a littermate from cage B from which they had been separated for 24 h. After 24 h (when CNO is no longer active), we performed a three-chamber task in which the subject mouse from cage A must discriminate between a cage B littermate and a novel mouse. Any increased exploration time of the novel mouse versus the littermate provides an index of social recognition memory (Fig. 4a). Despite the period of dCA2 silencing in *Amigo2*-Cre mice, both WT and *Amigo2*-Cre mice spent significantly more time interacting with the novel animal compared to the littermate (Fig. 4b, c). Thus, we conclude that continuous dCA2 activity is not needed to maintain a well-consolidated memory. Rather, the deficit in memory when dCA2 is silenced after encoding of a new social memory in trial 1 reflects a specific requirement for dCA2 activity in consolidation.

### dCA2 is needed for recall of a consolidated social memory

Conventional lesion studies leading to chronic loss of HPC indicate that fully consolidated long-term memories are ultimately stored in neocortex, where their recall becomes largely independent of HPC. However, acute silencing of HPC using optogenetics was found more recently to impair recall of remote long-term memory^20^, implying that long-term silencing of HPC may trigger changes in other brain regions that allow them to compensate for the loss of HPC during recall. To assess if dCA2 is required for recall of a well-consolidated social memory, we examined the effect of acute silencing of dCA2 immediately prior to the three-chamber test. *Amigo2*-Cre mice and WT littermates previously injected with AAV-DIO-hM4Di-IRES-mCitrine were both given CNO IP 30 min prior to the social discrimination trial (Fig. 4d). As expected, WT mice spent more time interacting with a novel animal compared to a littermate. In contrast, *Amigo2*-Cre mice failed to discriminate between the two mice (Fig. 4e, f), indicative of impaired recall of social memory. Thus, our data show that whereas dCA2 silencing does not disrupt the stability of a long-term, well-consolidated social memory, dCA2 activity is required for memory recall. Next, we address the circuit mechanisms whereby dCA2 participates in social memory.

### dCA2 targets a vCA1 region that projects to the NAc shell

Recently, Okuyama et al.^10^ reported that vCA1 PNs are necessary for social memory by actively storing social engrams. Furthermore, Okuyama et al.^10^ found that social memory was mediated by the projections from vCA1 PNs to the shell of the NAc. More recently, vCA3 PNs, which provide input to vCA1, were also implicated in social memory^11^. What is the nature of the link between the roles of dCA2 and vCA1/vCA3 in social memory? Do they reflect a single circuit in series, with dCA2 providing key information to vHPC, or are there separate parallel but necessary paths for social memory? Although dCA2 cells have been reported to project to vHPC^10,12^, their functional effects have not been explored. We therefore carried out a detailed anatomical and electrophysiological analysis of these projections and examined their potential role in social memory.

**Fig. 5:**
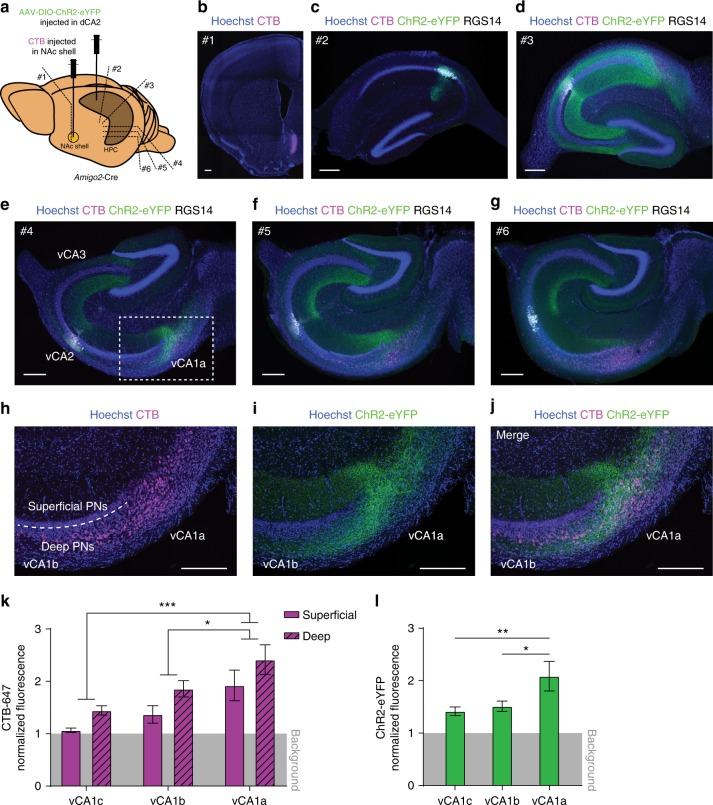
dCA2 targets a vCA1 site containing PNs that project to the NAc shell. **a** AAV-DIO-ChR2-eYFP was injected in dCA2 and the retrograde label CTB-647 was injected into the NAc shell of *Amigo2*-Cre mice. Approximate location of sections in **b**, **c**, **d**, **e**, **f**, and **g** are indicated (position #1, #2, #3, #4, #5, and #6, respectively). **b** Site of injection of CTB-647 (magenta). **c** Dorsal HPC section showing ChR2-eYFP (green) expression in dCA2 PN cell bodies (co-expressing RGS14, white) with no CTB-647 staining (lack of magenta). **d** Transverse section of intermediate HPC showing dCA2 fibers (labeled with ChR2-eYFP, green) distributed throughout CA3 and CA1 and sparse CTB-647 labeling (magenta). **e**–**g** Transverse sections progressing through vHPC showing dCA2 fibers (green) in vCA3, distal vCA1 (vCA1a) and adjacent ventral subiculum. Retrograde CTB-647 labeling of projections to NAc shell is seen in deep distal vCA1 (vCA1a) and adjacent ventral subiculum (magenta). **h**–**j** High magnification views of dotted area in **e**. **k** Quantification of normalized CTB-647 fluorescence in deep versus superficial PNs cell layers in vCA1c, vCA1b, and vCA1a. Ventral CA1 fluorescence was greater in deep versus superficial layers (*n* = 9 slices, 3 mice, repeated measures two-way ANOVA: deep/superficial *F*(1,8) = 55.05, *P* < 0.0001). Fluorescence was greater in distal (vCA1a) versus more proximal (vCA1b and vCA1c) areas (*n* = 9 slices, 3 mice, repeated measures two-way ANOVA: proximal-distal *F*(2,16) = 14.96, *P* = 0.0002, vCA1a vs vCA1b *P* = 0.0135, vCA1a vs vCA1c *P* = 0.0002, vCA1b vs vCA1c *P* = 0.1404, Tukey’s multiple comparisons test). **l** Quantification of normalized fluorescence intensity of ChR2-eYFP signal in dCA2 projections in vCA1. Signal was more intense in vCA1a compared to vCA1b and vCA1c (*n* = 9 slices, 3 mice, repeated measures one-way ANOVA: *F*(2,16) = 7.058, *P* = 0.0063, vCA1a vs vCA1b *P* = 0.0231, vCA1a vs vCA1c *P* = 0.0083, vCA1b vs vCA1c *P* = 0.8702, Tukey’s multiple comparisons test). Results in **k** and **l** show mean ± s.e.m. *P < 0.05; **P < 0.01; ***P < 0.001. Scale bars, 250 µm

We traced dCA2 PNs axons by bilaterally injecting into dCA2 of *Amigo2*-Cre mice a Cre-dependent virus rAAV2/5 EF1a.DIO.hChR2(E123T/T159C)-eYFP (AAV-DIO-ChR2-eYFP), expressing channelrhodopsin-2 tagged with eYFP (ChR2-eYFP). To determine whether there is overlap between vCA1 targets of dCA2 and the location of vCA1 PNs that project to the NAc shell, three weeks after viral injection into dCA2 we unilaterally injected into the NAc shell the retrograde tracer cholera toxin subunit B conjugated with Alexa Fluor 647 (CTB-647) (*n* = 3 mice) (Fig. 5a, b). One week after CTB-647 injection, the HPC was dissected and the pattern of labeling examined in transverse hippocampal sections.

In dorsal HPC (dHPC) (first third of the longitudinal axis), nearly all neurons that expressed ChR2-eYFP were identified as CA2 PNs, based on co-expression of RGS14 (Fig. 5c; 95.57 ± 1.12% overlap). Moreover, the majority of CA2 PNs at the site of viral injection expressed ChR2-eYFP (78.57 ± 2.67% of RGS14-positive cells, *n* = 6 mice). Intermediate HPC slices (the middle third of the HPC), where no CA2 PNs were infected by virus, received dense projections from dCA2 throughout the CA3 and CA1 regions (Fig. 5d). In vHPC (last third of HPC) we observed relatively few dCA2 fibers in vCA3. Along the vCA3 transverse axis, dCA2 fibers were preferentially located in vCA3c (the subregion closest to DG) compared to vCA3b and vCA3a (middle of CA3 and region closest to CA2, respectively) (Fig. 5e–g and Supplementary Fig. 6). In contrast, vCA1 received a strong projection from dCA2 that was densest in vCA1a (the area near the subiculum) and adjacent ventral subiculum (Fig. 5e–g, i, j, l). Of particular interest, vCA1a corresponded to the region of vCA1 that projected to the NAc shell, based on retrograde labeling with CTB-647 (Fig. 5e–g, h, j, k). Furthermore, as also reported by Okuyama et al.^10^, the vCA1 neurons that project to the NAc shell are mainly located in the deep layer of stratum pyramidale (closest to stratum oriens along the deep-superficial radial axis). No CTB-647 labeled cells were found in the ipsilateral dHPC (Fig. 5c) or contralateral HPC (Supplementary Fig. 7). Thus, we confirmed that dCA2 does indeed project to vHPC and targets preferentially the vCA1 region that projects to the NAc shell (vCA1a), suggesting a potential dCA2→vCA1a→NAc circuit for social memory storage and readout.

### dCA2 provides strong excitatory drive to vCA1a PNs

**Fig. 6:**
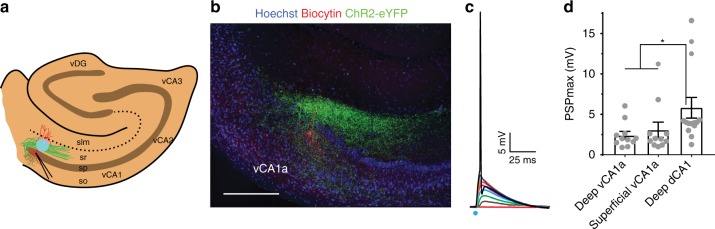
dCA2 provides excitatory input to vCA1a PNs. **a** Diagram of patch-clamp recordings from vCA1 PNs in response to photostimulation of ChR2-eYFP-expressing fibers from dCA2 PNs. Typical recording site in vCA1a. **b** Post hoc immunohistochemistry after patch-clamp recording from a vCA1a PN showing the recorded cell (filled with biocytin, red) and ChR2-eYFP-labeled dCA2 fibers (green). **c** Photostimulation (1 ms blue light pulse) of dCA2 inputs with increasing strengths of illumination evoked PSPs of increasing amplitude in a vCA1a PN. Highest intensity light pulse elicited an action potential. **d** Peak PSP depolarization from deep and superficial vCA1 PNs and from deep dCA1 PNs in response to photostimulation of dCA2 inputs at maximum light intensity (light intensity before action potentials were triggered or beyond which no further increase in PSP amplitude was observed). No difference was found between deep and superficial vCA1 PNs PSPs (*n*_deep vCA1a_ = 11 cells, 6 mice; *n*_superficial CA1a_ = 11 cells, 7 mice; unpaired *t* test: *t*(20) = 0.6334, *P* = 0.5337). Deep dCA1 PNs PSPs (*n* *=* 14 cells, 7 mice) were ~twofold larger in amplitude than those in vCA1 (deep and superficial combined; unpaired *t* test: *t*(34) = 2.540, *P* = 0.0158). Results show mean ± s.e.m. *P < 0.05. Circles represent individual cells. Scale bar, 250 µm (**b**)

To explore whether the projections from dCA2 to vCA1 form functional synapses, we injected the dCA2 region of *Amigo2*-Cre mice with AAV-DIO-ChR2-eYFP and, after 4 weeks, obtained acute slices from vHPC for electrophysiological recordings. Postsynaptic potentials (PSPs) elicited by photostimulation of ChR2-eYFP expressed in fibers from dCA2 were examined with whole-cell patch-clamp recordings from vCA1 and vCA3 PNs (Fig. 6a). A single 1-ms blue light pulse reliably elicited a net depolarizing PSP in about 90% of vCA1a PNs (22/24 cells; Fig. 6b, c) that was sufficient to elicit an action potential (with strong illumination) in about 30% of those (7/22 cells). Similar photostimulation of dCA2 inputs failed to evoke a detectable synaptic response in vCA1b or vCA1c PNs in the same slices where depolarizing PSPs were found in vCA1a (vCA1b: *n* = 5 cells, 3 mice; vCA1c: *n* = 3 cells, 3 mice). Nor did we observe responses in vCA3 (*n* = 8 cells, 3 mice), although post hoc immunohistochemistry confirmed the presence of dCA2 fibers in vCA3. In contrast to the findings that dCA2 produces a ~threefold larger EPSP in deep compared to superficial dorsal CA1 (dCA1) PNs^21^, we found no significant difference in the magnitude of the PSP elicited by dCA2 inputs in deep compared to superficial vCA1a PNs using maximal intensity photostimulation, although the magnitude of the dCA2-evoked PSP in vCA1a was approximately half that in deep dCA1 PNs (Fig. 6d).

**Fig. 7:**
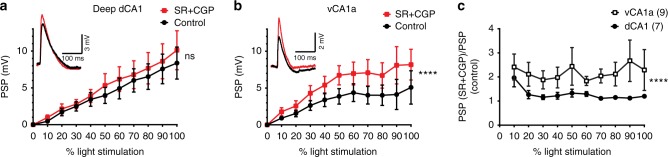
dCA2 inputs recruit more disynaptic inhibition in vCA1a than in deep dCA1. AAV-DIO-ChR2-eYFP was injected in dCA2 of *Amigo2*-Cre mice. **a** Synaptic input-output curves following photostimulation of dCA2 inputs at indicated intensities in deep dCA1 PNs, before and after blockade of GABA_A_ receptors with 2 µM SR 95531 and GABA_B_ receptors with 1 µM CGP 55845. 100% light intensity defined in Fig. 6 legend. Blockade of inhibition caused a small, statistically non-significant increase in the peak PSP (*n* = 7 cells, 3 mice; two-way ANOVA: treatment × light intensity *F*(10,132) = 0.06631, *P* > 0.9999; treatment *F*(1,132) = 1,741, *P* = 0.1893). **b** Input-output curves following photostimulation of dCA2 inputs to vCA1a PNs, before and after 2 µM SR 95531 and 1 µM CGP 55845. Blockade of inhibition blockade produced a significant ~twofold increase in the PSP amplitude (*n* = 9 cells, 5 mice; two-way ANOVA: treatment × light intensity *F*(10,154) = 0.4744, *P* = 0.9046; treatment *F*(1,154) = 17.79, *P* < 0.0001). **c** Ratio of the peak PSP after inhibition block  divided by PSP before inhibition block is greater in vCA1a than in deep dCA1 (two-way ANOVA: dorsal/ventral × light intensity *F*(9,129) = 0.2679, *P* = 0.9821; dorsal/ventral *F*(1,129) = 23.06, *P* < 0.0001). Results show mean ± s.e.m.****P < 0.0001; ns, *P* > 0.05

At least part of the reason for the smaller dCA2-evoked net PSP in vCA1a compared to deep dCA1 PNs was due to a greater amount of feed-forward inhibition in vCA1a. Thus, blockade of GABA_A_ and GABA_B_ receptors with 2 µM SR 95531 and 1 µM CGP 55845, respectively, caused only a small, statistically non-significant increase in the peak PSP depolarization of deep dCA1 PNs (Fig. 7a) but produced a significant ~twofold increase in the PSP amplitude of vCA1a cells (Fig. 7b, c). The dCA2-evoked EPSP measured in the presence of the GABA blockers was similar in size in deep dCA1 compared to vCA1a PNs, indicating similar extents of excitatory drive. Thus, dCA2 sends a functional excitatory projection to vCA1a that is capable of eliciting spike output in response to a single brief stimulus, despite increased disynaptic inhibition relative to that evoked by dCA2 input to dCA1.

### dCA2-to-vHPC projections are necessary for social memory

**Fig. 8:**
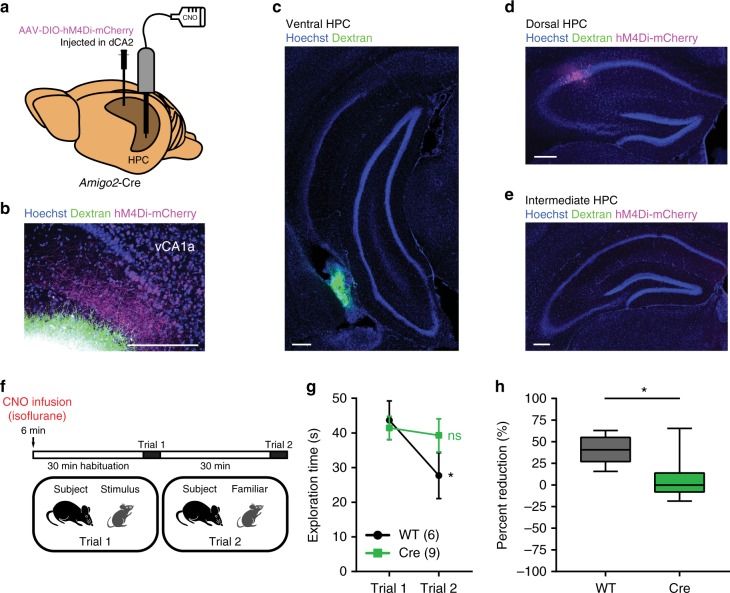
dCA2 projections to vHPC are necessary for social memory. **a** AAV-DIO-hM4Di-mCherry was injected in dCA2 of *Amigo2*-Cre mice and WT littermates. A cannula guide was implanted for local CNO or dye infusion in vCA1. **b** Ventral HPC horizontal slice showing overlap between dCA2 fibers located in vCA1a (labeled with hM4Di-mCherry, magenta) and dextran conjugated to Alexa Fluor 680 (green) infused in vCA1. **c** Ventral HPC coronal slice from an *Amigo2*-Cre mouse after infusion in vHPC of a dextran conjugated to Alexa Fluor 488 (green). **d** Dorsal HPC coronal slice from same brain as in **c**, showing expression of hM4Di-mCherry (magenta) but no Alexa signal. **e** Intermediate HPC coronal slice showing lack of signal for hM4Di-mCherry or Alexa dye. **f** A direct interaction test was performed 30 min after bilateral local infusion of CNO in vHPC (2 µL of 1 mM solution per side). **g** WT mice displayed decreased social exploration time during trial 2 (*n* = 6, *P* = 0.0110, Sidak’s multiple comparisons test) whereas hM4Di-mCherry expressing *Amigo2*-Cre mice showed no decrease (*n* *=* 9, *P* = 0.8351, Sidak’s multiple comparisons test). The two groups differed significantly (two-way ANOVA: treatment × trial *F*(1,13) = 4.964, *P* = 0.0442). **h** The percent reduction in interaction time in trial 2 versus trial 1 in *Amigo2*-Cre mice was significantly less than in WT mice (unpaired *t* test: *t*(13) = 2.962, *P* = 0.0110). Results in **g** show mean ± s.e.m. Box-whiskers plot in **h** present median (center line), extension from the 25th to 75th percentiles (box) and minimal and maximal values (whiskers). *P < 0.05; ns, *P* > 0.05. Scale bar, 250 µm (**b**, **c**, **d**, **e**)

Given that both dCA2 and vCA1 are necessary for social memory and that our anatomical and electrophysiological data show that dCA2 makes strong excitatory synaptic connections with vCA1a, we next investigated whether the dCA2 projections to vHPC are necessary for social memory. We injected in dCA2 of *Amigo2*-Cre mice and WT littermates rAAV2/8 hSyn.DIO.hM4D(Gi)-mCherry (AAV-DIO-hM4Di-mCherry), which allows for Cre-dependent expression of hM4Di tagged with mCherry (hM4Di-mCherry). A cannula guide was also implanted over vCA1 to allow local and selective delivery of CNO to achieve specific silencing of dCA2 projections to vHPC (Fig. 8a). We first examined the local targeting of this approach by infusing through the cannula Alexa Fluor 680 or Alexa Fluor 488 conjugated to dextran in animals expressing hM4Di-mCherry in dCA2. We waited ~30 min after dye infusion, then perfused and fixed the brains and then obtained coronal or horizontal brain slices along the dorsal–ventral axis of the HPC. Ventral HPC showed localized dye fluorescence at the site of infusion (Fig. 8b, c). In contrast, dHPC showed strong expression of hM4Di-mCherry in dCA2 PNs (97.18 ± 1.52% mCherry overlap with RGS14, with mCherry labeling 68.93 ± 8.31% of RGS14-positive cells near the site of injection, *n* = 6 mice) with no observable Alexa dye fluorescence (Fig. 8d). Intermediate HPC showed neither hM4Di-mCherry expression nor signal from the dye infused in vHPC (Fig. 8e). Thus, this approach allows us to selectively regulate dCA2 projections in vHPC.

Four weeks after bilateral viral injection into dCA2 and 1 week after cannula guide implantation, we performed the direct interaction test of social memory with a 30 min intertrial interval. Thirty minutes prior to trial 1, *Amigo2*-Cre and WT mice were anesthetized with isoflurane and CNO was bilaterally infused in vHPC through a cannula (Fig. 8f). The WT mice displayed intact social memory as evidenced by a decreased social exploration time during trial 2. In contrast, the *Amigo2*-Cre mice expressing hM4Di-mCherry showed no significant decrease in social exploration from trial 1 to trial 2, demonstrating impaired social memory (Fig. 8g, h).

To verify that CNO injected into vHPC was acting locally and did not diffuse to dHPC to silence dCA2 somatic output, we examined the effect of CNO infusion in vHPC on social aggression, which depends on AVPR1b expression in dCA2^22^. Our laboratory has found that silencing all dCA2 output by IP injection of CNO in mice expressing hM4Di in dCA2 was able to suppress aggression^23^. Thus, if ventral infusion of CNO suppressed social memory by diffusing to dCA2, it should also suppress aggression. However, we found that ventral infusion of CNO did not decrease aggression; rather there was a trend for aggression to increase (Supplementary Fig. 8). These results thus strongly suggest that CNO acted locally in vHPC and that the projections to vHPC from dCA2 are indeed required for social memory.

## Discussion

Our finding that acute chemogenetic or optogenetic silencing of dCA2 results in a deficit in social memory suggests that dCA2 actively participates in this form of declarative memory. This result is important as previous findings implicating dCA2 in social memory relied on chronic genetic silencing^6^ or chemical lesions^7^, which could have caused reactive changes in other brain regions (e.g., Boehringer et al.^9^). Further evidence supporting an active role of CA2 in social memory comes from extracellular in vivo electrophysiological recordings showing that CA2 PN place fields remap in response to novel or familiar conspecifics (as well as in response to novel objects)^24^. By taking advantage of the reversibility of acute manipulations and the dynamic properties of different social memory tasks, we found that dCA2 is necessary for all phases of social memory, including encoding, consolidation, and recall.

Our finding that dCA2 optogenetic silencing during memory encoding is sufficient to impair social memory complements a previous study showing that optogenetic activation of vasopressinergic inputs to dCA2 during encoding enhances the duration of social memory^8^. Moreover, our finding that inhibition of dCA2 for several hours soon after an encoding trial blocks social memory consolidation is consistent with the finding that infusion of a protein synthesis inhibitor in dHPC 3 h after social memory encoding is also able to block its consolidation^19^.

As recent results indicate that vCA1 PNs and their projections to the NAc shell are also necessary for social memory^10^, we examined whether dCA2 and vCA1 neurons are integrated into a single social memory circuit or whether they act through parallel circuits that are both required for social memory. Our findings that dCA2 PNs project to and excite vCA1 PNs and that projections to vHPC are necessary for social memory suggest that dCA2 provides important social information to vCA1 to support social memory. Of particular interest, dCA2 preferentially targets the subregion of vCA1 (vCA1a) that has been implicated in social memory behavior through its output to the NAc shell. Although dCA2 also sends anatomical projections to vCA3, which was recently shown to also participate in social memory storage^11^, we failed to observe functional synapses between dCA2 and vCA3, suggesting that vCA1 is the main target of dCA2 in vHPC. Thus, our results suggest that social memory information flows from dCA2 to vCA1a to the NAc shell in a single serial circuit, with vCA3 inputs to vCA1 providing a parallel but necessary path. Since vCA3 is not necessary for social memory recall^11^, whereas Okuyama et al.^10^ and our findings show, respectively, that vCA1 and dCA2 are important for recall, it is likely that vCA1 participates in social memory retrieval as a result of its input from dCA2, rather than from vCA3.

Further evidence implicating dCA2 in social memory comes from studies of the effects of the social neuropeptides oxytocin and arginine vasopressin (AVP). Two recent reports found that oxytocin receptors in the dCA2 and dorsal CA3a (dCA3a) areas of mice are necessary for the discrimination of social stimuli^25^ and for the persistence of long-term social memory^26^. Furthermore, in agreement with our findings, optogenetic terminal-specific silencing of dCA2/dCA3a projections to vCA1 impaired social recognition, whereas silencing of dCA2/dCA3a projections to dCA1 did not^4^. Although these studies did not use a genetic approach to selectively target CA2, and so could not distinguish the contributions of dCA2 versus neighboring dCA3a, the finding of Chiang et al.^11^ that selective silencing of dCA3 did not interfere with social memory implies that the effects of joint manipulations of dCA2/dCA3a on social memory likely reflect the contribution of dCA2. Interestingly, although oxytocin receptors in dCA2/dCA3a are not necessary for non-social stimuli discrimination, Raam et al.^25^found that dCA2/dCA3a projections to dCA1 are important for object discrimination, consistent with the role of dHPC in object recognition^27,7^. As selective silencing of dCA2 does not impair novel object recognition^6^, we hypothesize that a dCA3a→dCA1 pathway may participate in memory for non-social stimuli, whereas the dCA2→vCA1a→NAc pathway mediates social memory.

In addition to the importance of oxytocin receptors, AVP signaling through the AVPR1b receptor has been implicated in social behavior. Thus, a mouse line with a general deletion of the AVPR1b receptor, which is strongly expressed in CA2 PNs^29^, has impairments in social memory and social aggression^22,30–32^. In addition, as discussed above, Smith et al.^8^ found that optogenetic release of AVP in area dCA2 acts to enhance social memory encoding. In contrast, AVP release during the recall trial did not improve memory performance.

What is the nature of the social information that dCA2 provides to vCA1 for social memory storage? It is possible that dCA2 may encode a representation, or engram^33^, of social memory for a given stimulus mouse, similar to what is observed in vCA1^10^. Alternatively, dCA2 may provide a less specific signal of social novelty or familiarity, which may be integrated in vCA1 with individual-specific information, perhaps from vCA3^11^, to encode a rich representation of a conspecific. Although in vivo electrophysiological recordings have provided evidence that dCA2 neurons alter their firing in response to a novel or familiar mouse^24^, to date, no individual mouse-specific firing patterns (i.e., “Jennifer Aniston cells”^34^) have been observed.

Our work integrates dCA2 into the dCA2→vCA1→NAc circuit. As all three regions have been implicated in neuropsychiatric disorders^35–38^, this circuit likely represents an important disease-causing locus. Given the distinct molecular expression profile of CA2, modulation of CA2 activity represents an attractive potential target for the development of novel therapeutic approaches to such diseases.

## Methods

### Mice

All animal procedures were approved by the Columbia University and New York State Psychiatric Institute IACUC committees. We used 2–6-month-old grouped-housed *Amigo2*-Cre-positive (heterozygous) and Cre-negative (WT) littermates (3–5 per cage). The *Amigo2*-Cre line was maintained as a hemizygous line on the C57BL/6J background. In the direct interaction test, 4–5-week-old C57BL/6J juveniles were used as stimulus mice; in the three-chamber apparatus tests, C57BL/6J age-matched novel stimulus mice were used. Age-matched Balb/cJ mice were used as stimulus intruders in the resident–intruder paradigm. Behavior was performed on sexually naive male mice only. All animals were maintained on a 12-h light/dark cycle with ad libitum access to food and water.

### Surgical procedures

During surgeries, mice were maintained under isoflurane anesthesia at 1.5–5% and given analgesics. For viral injections, a craniotomy was performed above the target region and a glass pipette was stereotaxically lowered down the desired depth, using bregma as reference. Injections were performed using a nano-inject II (Drummond Scientific company). Aliquots of 23 nl were injected 15 s apart until the total amount was reached. The pipette was retracted after 10 min. Mice were used for experiments 3 weeks or more after viral injections.

For chemogenetic silencing of dCA2 for both behavior and electrophysiology experiments, we bilaterally injected into the HPC of *Amigo2*-Cre and WT littermates 300 nl of the virus rAAV2/2 hSyn.DIO.HA-hM4D(Gi).IRES.mCitrine (Addgene cat#50455, packed by the Duke University Vector Core, which we refer to as AAV-DIO-hM4Di-IRES-mCitrine). The injection coordinates were: −1.6 mm anteroposterior (AP), ±1.6 mm mediolateral (ML), and −1.7 mm dorsoventral (DV). For optogenetic silencing of dCA2, we bilaterally injected 200 nl of the virus rAAV2/5 EF1a.DIO.eArch3.0-eYFP (University of North Carolina Vector Core, which we refer to as AAV-DIO-eArch3.0-eYFP) using the following coordinates: −2.0 mm AP, ±1.8 mm ML, −1.7 mm DV. For silencing of dCA2-to-vHPC projections, we bilaterally injected 200 nl of the virus rAAV2/8 hSyn.DIO.hM4D(Gi)-mCherry (Addgene cat#44362; AAV-DIO-hM4Di-mCherry) using the following injection coordinates: −2.0 mm AP, ±1.8 mm ML, −1.7 mm DV. For the retrograde tracing from the NAc shell, 70 nl of CTB conjugated to Alexa Fluor 647 (ThermoFisher Scientific, cat#C34778) was unilaterally injected into the NAc shell using the following coordinates: +1.4 mm AP, 0.5 mm ML, −4.7 mm DV. For the anterograde tracing and electrophysiology studies of the dCA2-to-vCA1 projections, we injected bilaterally 200 nl of the rAAV2/5 EF1a.DIO.hChR2(E123T/T159C)-eYFP virus (University of North Carolina Vector Core cat#35509; AAV-DIO-ChR2-eYFP) into HPC of *Amigo2*-Cre mice using the following injection coordinates: −2.0 mm AP, ±1.8 mm ML, −1.7 mm DV.

For silencing of dCA2-to-vHPC projections, three weeks after injection of AAV-DIO-hM4Di-mCherry, mice were implanted with a cannula guide extending for 4 mm (Plastics One, cat#C315G 2-G11-SPC) below the pedestal. The scalp was removed and holes were drilled (−3.2 mm AP, ±3.2 mm ML). Cannula guides were kept in place using super-glue and dental cement (GC FujiCEM 2) and dummy cannulas (Plastics One, cat#C315DC-SPC) were inserted into the guides.

To confirm the position of the cannula guide and local volume of tissue that is targeted, 2 µL of dye (dextran conjugated to Alexa Fluor 680—cat#D34680—or Alexa Fluor 488—cat#D22910) was infused per site through the cannulas.

For testing the efficacy of optogenetics in silencing dCA2 PNs, a multimode optic fiber with a 100 µm core and a multishank silicon probe electrode array were implanted attached together (−2.0 mm AP, ±2.0 mm ML, −1.2 mm DV) and kept in place using dental cement (C&B-Metabond). The combined assembly was mounted in a movable microdrive for precise targeting of final coordinates. For optogenetic behavior experiments, only optic fibers (200 µm core) were bilaterally implanted using the same coordinates.

### Immunohistochemistry

Animals were perfused intracardially using saline followed by 4% PFA in PBS. The brains were quickly extracted and incubated in 4% PFA overnight. After 1 h washing in 0.3% glycine in PBS, 60 µm coronal or horizontal brain sections or 100 µm transverse sections of dissected HPC were prepared using a Leica VT1000S vibratome. The sections were permeabilized and blocked for 2 h with 0.4% Triton-X and 5% goat serum in PBS at room temperature and then incubated overnight with primary antibodies at 4 °C diluted in 0.1% Triton-X and 5% goat serum in PBS. The sections were washed three times for 15 min in PBS and goat secondary antibodies diluted at 1:500 were applied at room temperature for 3 h in 0.1% Triton-X and 5% goat serum in PBS. The sections were washed again three times for 15 min in PBS. Hoechst 33342 (ThermoFisher Scientific, cat#62249) staining was performed by applying 1:1000 Hoechst solution in PBS for 10 min at room temperature prior to mounting the slices using fluoromount mounting medium (Sigma-Aldrich). Images were acquired using an inverted confocal microscope (Leica, LSM 700).

For mCitrine and eYFP labeling, primary incubation was performed with chicken antibody against GFP (1:1000, AVES Labs, cat#GFP-1020) and secondary incubation was performed with antibody conjugated to Alexa Fluor 488 (ThermoFisher Scientific, cat#A11039). For RGS14 labeling, primary incubation was performed with mouse IgG2a antibody against RGS14 (1:500, UC Davis/NIH NeuroMab Facility, cat#75-170) and secondary incubation was performed with antibody conjugated to Alexa Fluor 488 (ThermoFisher, Scientific cat#A21131), Alexa Fluor 555 (ThermoFisher Scientific cat#A21137), or Alexa Fluor 647 (ThermoFisher Scientific, cat#A21241). For Nissl co-staining, slices were incubated with Neurotrace 640/660 (1:200, ThermoFisher Scientific, cat#N21483) together with secondary incubations used above. For post hoc immunocytochemistry after patch-clamp recordings with biocytin-filled electrodes, slices were first fixed for 1 h in 4% PFA in PBS. Streptavidin conjugated to Alexa Fluor 647 (1:500, ThermoFisher Scientific, #S21374) and primary antibody against GFP conjugated to Alexa Fluor 488 (1:500, ThermoFisher Scientific, #A21311) were applied overnight at 4 °C.

### Analysis of viral expression in CA2

To confirm proper targeted expression in CA2 of the different Cre-dependent AAVs used in these experiments, we analyzed the overlap between an expressed viral fluorescent marker and the CA2-specific marker RGS14. Images were acquired on a single focal plane using a ×20 objective centered on the dCA2 subregion.

### Quantitative analysis of pathway tracing

AAV-DIO-ChR2-eYFP was bilaterally injected in dCA2 of *Amigo2*-Cre mice and three weeks later the retrograde tracer CTB conjugated to Alexa Fluor 647 was unilaterally injected in the NAc shell. After an additional week, mice were perfused intracardially and their brains extracted and treated as described above. The HPC was dissected, placed upright into a 4% agarose mold and sliced into 100 μm slices with a Leica VT1000S vibratome. Slices from each third of HPC along its longitudinal axis (dorsal, intermediate, and ventral) were stained for eYFP and RGS14 and imaged on a single centered focal plane using a ×5 objective. Three ventral HPC slices (from the first, middle, and last third of ventral HPC) were used for quantification of ChR2-eYFP and CTB-Alexa Fluor 647 signals in different ventral HPC subregions. Areas vCA3 and vCA1 were subdivided into three equal-length subregions (c, b, and a) along the proximal to distal transverse axis (with CA3c closest to DG and CA1c closest to CA2). Fluorescence intensity of ChR2-eYFP was measured within each area of interest including stratum radiatum, stratum pyramidale, and stratum oriens; fluorescence intensity of CTB was measured in deep and superficial CA1 pyramidal cell layers along the radial axis. Superficial CA1 was defined as the dense inner layer of stratum pyramidale bordering stratum radiatum, roughly 30–40 μm. Deep CA1 included the rest of the cell body layer extending to stratum oriens. Background ChR2-eYFP fluorescence was measured in stratum lacunosum moleculare of vCA1; background CTB-647 fluorescence was measured in stratum pyramidal of vCA3. Normalized fluorescence was calculated by dividing fluorescence of each region of interest by the background fluorescence. Fluorescence intensity of each region was measured using *Fiji* software.

### In vitro electrophysiology

We prepared transverse hippocampal slices from 9-week-old to 12-week-old male mice. Animals were killed under isoflurane anesthesia by perfusion into the left ventricle of ice-cold solution containing the following: 10 mM NaCl, 195 mM sucrose, 2.5 mM KCl, 10 mM glucose, 25 mM NaHCO_3_, 1.25 mM NaH_2_PO_4_, 7 mM Na Pyruvate, 1.25 mM CaCl_2_, and 0.5 mM MgCl_2_. The HPC was removed in the same dissecting solution, placed upright into a 4% agar mold, and cut into 400 μm slices with a vibratome (VT1200S, Leica) in the same ice-cold dissection solution. Slices were then transferred to a chamber containing 50% dissecting solution and 50% ACSF (125 mM NaCl, 2.5 mM KCl, 22.5 mM glucose, 25 mM NaHCO_3_, 1.25 mM NaH_2_PO_4_, 3 mM Na Pyruvate, 1 mM Ascorbic acid, 2 mM CaCl_2_, and 1 mM MgCl_2_). The chamber was kept at 34 °C for 30 min and then at room temperature for at least 1 h before recordings, which were performed at 33 °C. Dissecting and recording solutions were both saturated with 95% O_2_ and 5% CO_2_, pH 7.4. Slices were mounted in the recording chamber under a microscope.

Recordings were acquired using a Multiclamp 700 A amplifier (Molecular Device), data acquisition interface ITC-18 (Instrutech), and the Axograph X software. We targeted dCA2 and vCA1 PNs based on somatic location and size in both deep and superficial layers. Whole-cell recordings were obtained from either dCA2 or vCA1 PNs in current-clamp mode with a patch pipette (3–5 MΩ) containing the following: 135 mM K methylsulfate, 5 mM KCl, 0.2 mM EGTA-Na, 10 mM HEPES, 2 mM NaCl, 5 mM ATP, 0.4 mM GTP, 10 phosphocreatine, and 5 μM biocytin, pH 7.2 (280–290 mOsm). The liquid junction potential was 1.2 mV and was uncorrected. Series resistance (15–25 MΩ) was monitored throughout each experiment; cells with a >20% change in series resistance were discarded. Once whole-cell recording was achieved we confirmed the cell-type based on its electrophysiological properties. Rheobase was defined as the minimal current amplitude required for firing an action potential and was measured before and 15 min after CNO (Tocris, #4936) application to the bath solution (1 mM). For photostimulation, single pulses of blue light (pE-100, Cool LED) lasting 1 ms were delivered through a ×40 immersion objective and illuminated an area of 0.2 mm^2^. In a subset of experiments, GABA_A_ and GABA_B_ receptors were blocked with SR 95531 (2 μM, Tocris #1262) and CGP 55845 (1 μM, Tocris #1248), respectively.

### Optic fiber preparation

Multimode fibers with 100 μm or 200 μm cores were used (0.39 numerical aperture), respectively for in vivo electrophysiology recordings and behavior experiments. The acrylate jacket was removed, fibers were cut to ~5 cm, stripped at one end (~1 cm) and glued to a ceramic ferrule. All fibers were polished at the ferrule side to enhance coupling efficiency, which was determined by measuring the light power emitted from the fiber tip of the coupler patch cord using a power meter. Fibers emitting >80% of the light power entering the coupler were used for the experiments.

### In vivo electrophysiology

Green 1-s light pulses (539 nm) at 5 mW were used to assess the change in firing rate of dCA2 PNs expressing eArch3.0-eYFP compared to baseline. Electrodes were connected to an amplifier board (RHD214) and after that to an interface board (RHD2000) which allowed us to record broad band electrophysiological signals. Signals were acquired at 20 KHz and single cells were later obtained from the high-pass band of these signals (>500 Hz), sorted offline with a template-based clustering algorithm (Kilosort) and manually curated using Phy GUI.

### Behavioral tests

Mice were habituated to handling for 5 days before behavioral tests were begun. When appropriate, mice were also habituated to IP injections with daily IP injections of 0.1 ml of saline. Subject mice were not reused in the same behavior paradigm, with the exception for an optogenetic control experiment (no light pulses) with *Amigo2*-Cre mice. We waited a minimum of 1 week between experiments. Subject and stimulus mice were habituated to the behavioral room for 30 min before behavioral testing. *Amigo2*-Cre mice injected with Cre-dependent AAV expressing GFP or WT littermates (Cre-negative) injected with AAV-DIO-hM4Di-IRES-mCitrine, AAV-DIO-hM4Di-mCherry, or AAV-DIO-eArch3.0-eYFP were used as control groups. All apparatuses and testing chambers were cleaned with 70% propan-2-ol wipes (VWR) before each test. Behavior tasks were performed shortly after the beginning of the dark cycle in an observation room under red light and recorded with an overhead FireWire camera (DMK 31AF03-Z2; The Imaging Source) and ANY-maze (Stoelting). For IP injections, CNO was injected at 10 mg kg^−1^ (1 mg ml^−1^ solution in saline). For dCA2-to-vHPC projection silencing, mice were placed under a light isoflurane anesthesia (2%) and the dummy cannula was removed. A cannula (Plastics One, #C315I-SPC) projecting 0.8 mm from the tip of the cannula guide was mounted. Two µL of a 1 mM CNO solution was infused per site over 2 min using a syringe pusher (Fusion 200, Chemix Inc.) mounted with a 2 µL syringe (Hamilton, #88511). The cannula was removed 1 min after the end of the micro-infusion to avoid pulling out the drug when removing the cannula. Subjects were then moved to the test cages and fully recovered from the light anesthesia within 5 min. For optogenetic silencing of dCA2, the optic fibers implanted above dCA2 were connected to a laser (539 nm wavelength at 5 mW) with a mating sleeve. A light pulse was applied at the start of the 2-min-long trial 1 of the direct interaction test and light intensity was progressively decreased at the end of trial 1 over a period of 5 s to avoid rebound excitation.

Following behavioral experiments, animals were transcardially perfused and the brains fixed for post hoc histological verification of viral expression. Viral expression was detected throughout the most anterior 1/3 of the dHPC from all mice except two, which were excluded from the analysis.

### Direct interaction test

The procedure was adapted from Kogan et al.^17^. Subject mice were placed into individual plastic test boxes with fresh bedding of similar area to their home cage (29 cm long × 16.5 cm wide × 19.5 cm high) for 30 min. A male juvenile stimulus mouse (4–5 weeks old) was placed into the test box for an initial interaction trial of 2 min and then removed. After an interval, the same or a novel juvenile stimulus mouse was placed back into the subject’s cage for the second 2 min interaction trial. The intertrial interval duration depended on the particular experiment. For experiments with a 30 min interval, the subject mice remained in the experimental box. When the intertrial interval lasted for 24 h, the subjects were placed into their home cage after trial 1 and the same habituation procedure was repeated for the second interaction trial. The duration of social exploration of the juvenile by the subject mice was later scored offline by a trained observer blind to the experimental condition using ANY-maze software. Sniffing of any part of the body, close following and allogrooming were scored as social exploration behaviors. Mice that were aggressive towards the juvenile (including social dominance behavior) or that interacted for less than 24 s during trial 1 were excluded from statistical analysis. Percent reduction was calculated by the difference in social exploration between the first and the second trials divided by the total time of social exploration during the first trial, multiplied by 100%.

### Three-chamber littermate recall and sociability tests

The three-chambered box was a rectangular apparatus (each chamber was 19 cm long × 40.5 cm wide × 22 cm high) made from clear Plexiglas, with openings (10 cm wide) allowing access into each chamber. The assay consisted of three trials. The subject mice were initially allowed to explore the three empty chambers freely for 10 min. After this initial habituation trial, when the subjects crossed the center chamber, the doorways into the two side chambers were closed and two empty round wire cups (10 cm high, with a bottom diameter of 10 cm and bars spaced 2 cm apart) were placed on the side chambers. A weighted cup was placed on the top of the wire cups to prevent the subject mouse from climbing on top of the cups. Both doors to the side chambers were then opened and the subject mice were allowed to explore the empty cups and the entire apparatus for 10 min. After exploration, the subject mice were confined again to the center chamber. Next, if social memory recall was being tested, a co-housed littermate and an aged-matched unfamiliar male stranger that had no prior contact with the subject mouse were each placed inside one of the wire cups. If sociability was being tested, a novel unfamiliar male was placed in one cup and the other was left empty. Finally, both doors to the side chambers were reopened and the subject was allowed to explore the entire apparatus for the 10 min long test trial. The time spent in each chamber and the total distance traveled were later automatically measured with ANY-maze software. Social discrimination and social preference scores were calculated as the difference between the time spent in the two side chambers divided by the total time spent in both chambers.

### Social aggression

The resident–intruder paradigm was adapted from Wersinger et al.^31^ to assess social aggression. Subject male mice (residents) were individually housed for 1 week, with no cage change prior to the encounter with a novel intruder. Stimulus mice (male Balb/cJ intruders) were grouped-housed and used for only a single encounter. Feeding and water apparatuses were removed before a 1-h habituation period to allow unimpeded interaction. Age-matched and weight-matched intruders were presented in the home cages of the resident mice for 10-min sessions. Attack was allowed to continue 2 min after its onset, which was defined by a bite. On the rare occasion that a stimulus mouse attacked the resident (*n* = 1 out of 33 mice), the trial was halted.

Ethological analysis of aggression was later performed by a blind observer, recording number of bites, number of attacks, attack duration, and number of tail rattles. These various behaviors are defined as follows: the initiation of an attack is defined by a clear bite initiated by the resident mouse. Attack bouts are cycles of initiated attack with continuous orientation and physical interaction by the resident towards the intruder. They are defined as completed when the resident has physically reoriented away from the intruder. “Attack duration” is the time spent biting, pursuing, mounting, and engaging in excessive allogrooming behavior. “Social dominance” excludes biting and is defined as mounting behavior or persistent face allogrooming.

### Statistical analysis

Prism 7 (GraphPad) was used for statistical analysis and to graph data. Statistical significance was assessed by two-tailed unpaired Student’s *t* test, two-tailed paired Student’s *t* test, two-way ANOVA, two-way repeated measures ANOVA, one-way repeated measures ANOVA with Geisser-Greenhouse correction or the Freeman–Halton extension of the Fisher’s exact probability test where appropriate, as described in the figure legends or main text. Post hoc analysis was conducted using Sidak’s multiple comparisons test or Tukey’s multiple comparisons test. Results were considered significant when *P* < 0.05. *α* was set equal to 0.05 for post hoc analysis tests. *P < 0.05, **P < 0.01, ***P < 0.001, ****P < 0.0001, and ns indicates a non-significant result (*P* > 0.05). Sample sizes were chosen on the basis of previous studies.

## Electronic supplementary material

Supplementary Information

## Data Availability

The authors declare that all data supporting the findings of this study are available within the paper and its supplementary information file.
