# Supplementary material for: A hippocampal circuit linking dorsal CA2 to ventral CA1 critical for social memory dynamics
Source: Nat Commun. 2018 Oct 9;9:4163. doi: 10.1038/s41467-018-06501-w (PMC6178349; doi:10.1038/s41467-018-06501-w)
Supplement: Supplementary file 1 — Supplementary Information [file 41467_2018_6501_MOESM1_ESM.pdf]

Supplementary information

A hippocampal circuit linking dorsal CA2 to ventral CA1 critical for social memory dynamics

Meira et al.

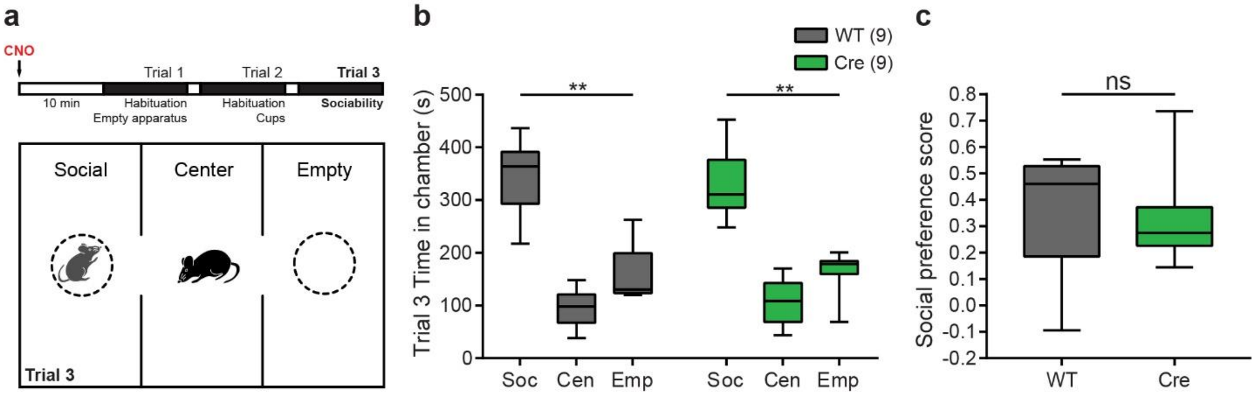

Supplementary Figure 1 | dCA2 acute silencing does not affect sociability.

(a) The three-chamber sociability test was performed in WT and *Amigo2*-Cre mice previously injected with AAV-DIO-hM4Di-IRES-mCitrine in dCA2. The task included two 10-min habituation trials (first to the empty apparatus, trial 1, then to two empty wire cups placed in opposite side chambers, trial 2) and a final sociability trial, in which a novel mouse is placed inside one of the cups (trial 3). Mice were injected with CNO IP 30 min prior to the sociability trial. (b) During the sociability trial, both the WT and the *Amigo2*-Cre mice spent more time in the novel animal chamber (Soc, social) compared to the empty cup (Emp) chamber (WT repeated measures one-way ANOVA: $n = 9$, $F(1.267, 10.13) = 35.2$, $P < 0.0001$, Social vs Empty $P = 0.0035$, Tukey's multiple comparisons test; Cre repeated measures ANOVA: $n = 9$, F (1.431, 11.45) = 32.33, $P < 0.0001$, Social vs Empty $P = 0.0023$, Tukey's multiple comparisons test). The groups did not differ significantly (two-way ANOVA: treatment x chamber $F(2,32) = 0.2359$, $P = 0.7912$). (c) No difference on social preference score was found between groups (unpaired t test: $t(16) = 0.4407$, $P = 0.6653$). Box-whiskers plots present median (center line), extension from the 25th to 75th percentiles (box) and minimal and maximal values (whiskers). **$P<0.01$; ns, $P>0.05$.

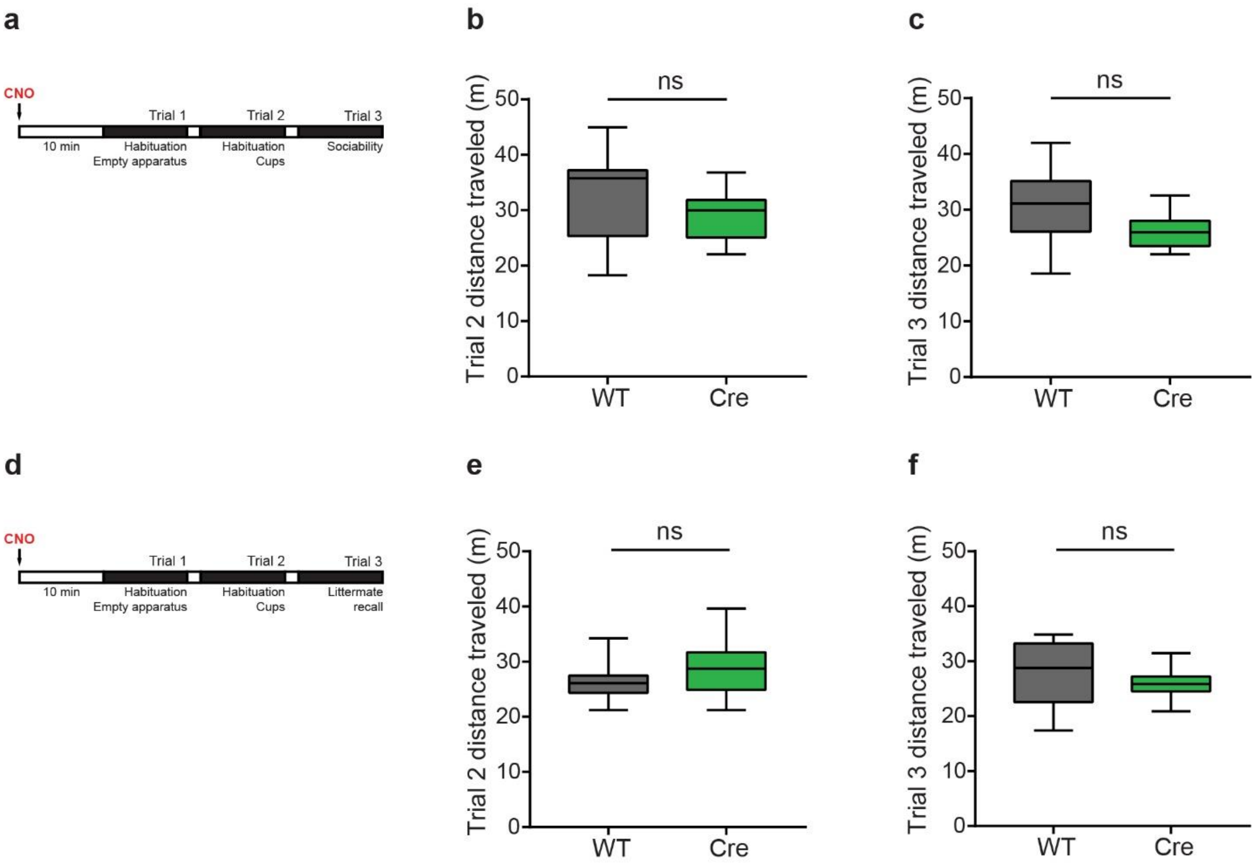

Supplementary Figure 2 | dCA2 acute silencing does not affect locomotion.

(a) Three-chamber sociability test protocol. (b) Locomotion during the last habituation trial (trial 2). (c) Locomotion during the sociability trial (trial 3). WT and the *Amigo2*-Cre mice traveled a similar distance (Trial 2 unpaired t test: $t(16) = 1.085$, $P = 0.2942$; Trial 3 unpaired t test: $t(16) = 1.758$, $P = 0.0978$). (d) Three-chamber social memory recall test protocol. Mice were habituated to empty arena (trial 1) and then to empty cups (trial 2). In trial 3, one cup contained a novel adult male and the other cup contained a male littermate. (e), (f) During trials 2 (e) and 3 (f), *Amigo2*-Cre and their WT littermates traveled a similar distance (Trial 2 unpaired t test: $t(17) = 1.156$, $P = 0.2636$; Trial 3 unpaired t test: $t(17) = 0.8087$, $P = 0.4298$). Mice had been injected in dCA2 with AAV-DIO-hM4Di-IRES-mCitrine in all experiments in this figure. Box-whiskers plot in h present median (center line), extension from the 25th to 75th percentiles (box) and minimal and maximal values (whiskers). ns, $P>0.05$.

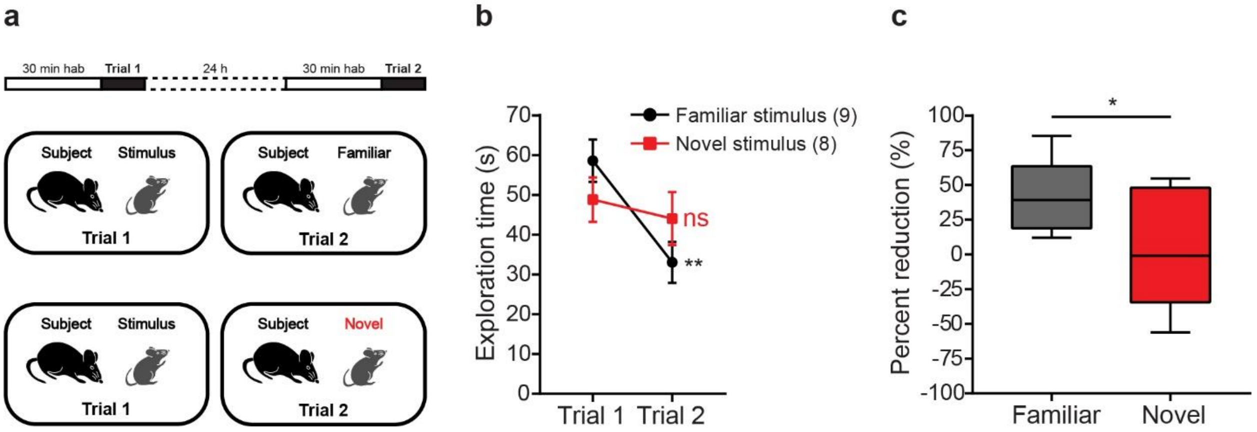

Supplementary Figure 3 | Direct interaction test with 24 h intertrial interval.

(a) WT mice performed the direct interaction test with a 24 h intertrial interval, using in trial 2 either the same mouse encountered on trial 1 (familiar) or a different (novel) stimulus mouse. (b) Only mice that reencountered the familiar stimulus mouse showed decreased social exploration time during trial 2 (Familiar stimulus: n = 9, P = 0.0032, Sidak's multiple comparisons test; Novel stimulus: n = 8, P = 0.7595, Sidak's multiple comparisons test). The groups differed significantly (two-way ANOVA: treatment x trial $F(1,15)$ = 4.58, P = 0.0492). (c) The group of mice which encountered a novel stimulus mouse in trial 2 had a lower percent reduction in social exploration compared to mice which met the same stimulus mouse on both trials (unpaired t test: $t(15)$ = 2.256, P = 0.0395). Results in b are mean ± s.e.m. Box-whiskers plot in c present median (center line), extension from the 25th to 75th percentiles (box) and minimal and maximal values (whiskers). *P<0.05; **P<0.01; ns, P>0.05.

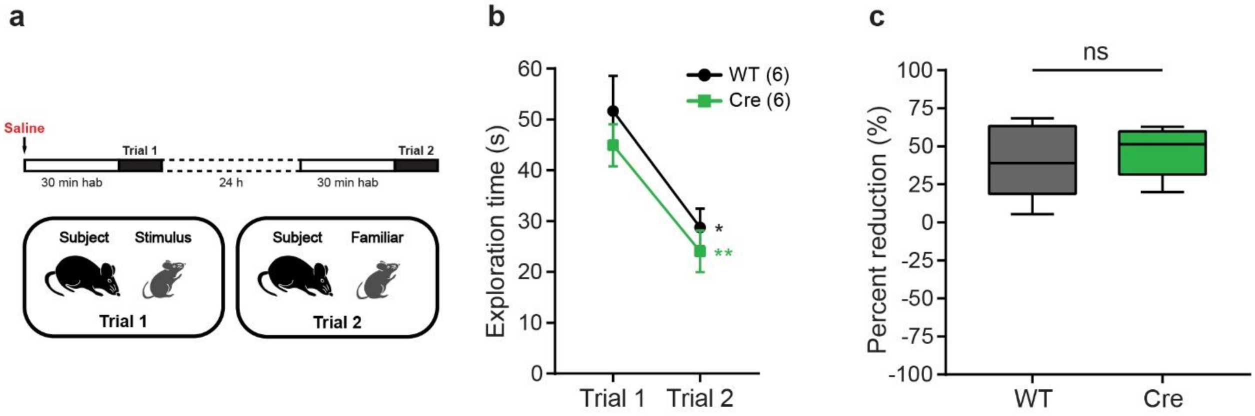

Supplementary Figure 4 | *Amigo2*-Cre background or hM4Di expression alone does not affect social memory.

(a) Protocol for control experiment examining effects of hM4Di expression in *Amigo2*-Cre mice on social memory. Saline (instead of CNO) was injected IP 30 min prior to the direct interaction test with 24 h intertrial interval. Both WT and *Amigo2*-Cre littermates were injected in dCA2 with AAV-DIO-hM4Di-IRES-mCitrine. (b) WT controls and *Amigo2*-Cre mice showed decreased social exploration time during trial 2 (WT: $n = 6$, $t(5) = 3.357$, $P = 0.0202$, paired t test; Cre: $n = 6$, $t(5) = 5.307$, $P = 0.0032$, paired t test). The groups did not differ significantly (two-way ANOVA: treatment x trial $F(1,10) = 0.06456$, $P = 0.8046$). (c) No difference was found in percent reduction in social exploration time in trial 2 compared to trial 1 between groups (unpaired t test: $t(10) = 0.5989$, $P = 0.5626$). Results in b show mean ± s.e.m. Box-whiskers plot in c present median (center line), 25th to 75th percentiles (box) and minimal and maximal values (whiskers). *$P<0.05$; **$P<0.01$; ns, $P>0.05$.

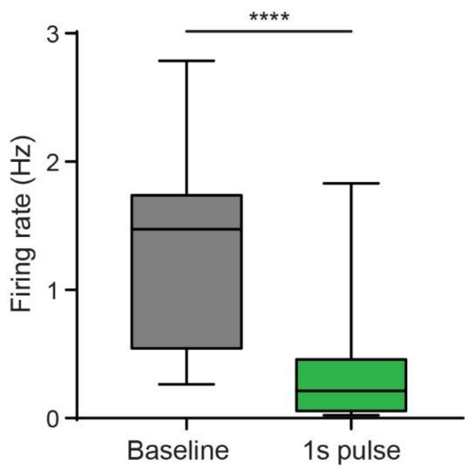

Supplementary Figure 5 | dCA2 PNs in vivo firing rate decreases in response to optogenetic silencing.

Amigo2-Cre mice were injected with AAV-DIO-eArch3.0-eYFP. CA2 PNs mean firing rate was recorded in vivo using silicon probe multielectrode array. Firing rates during 1-s long pulses of green light (5 mW at 539 nm) decreased firing rate of CA2 PNs compared to baseline firing rate (n = 24 cells, 3 mice, paired t test: t(23) = 9.563, P < 0.0001). Box-whiskers plot present median (center line), extension from the 25th to 75th percentiles (box) and minimal and maximal values (whiskers). ****P<0.0001.

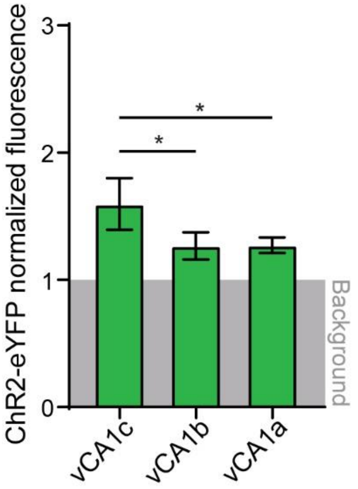

Supplementary Figure 6 | Quantification of dCA2 projections to vCA3.

(a) Quantification of normalized ChR2-eYFP fluorescence signal from *Amigo2*-Cre mice injected in dCA2 with AAV-DIO-ChR2-eYFP in areas vCA3c, vCA3b and vCA3a. Ventral vCA3c showed higher normalized fluorescence intensity than vCA3b and vCA3c (*n* = 9 slices, 3 mice, repeated measures one-way ANOVA: $F(2,16) = 5.388$, $P = 0.0163$, vCA3c vs vCA3b $P = 0.0286$, vCA3c vs vCA3a $P = 0.0314$, vCA3b vs vCA3a $P = 0.9988$, Tukey's multiple comparisons test). Results show mean ± s.e.m. *P<0.05.

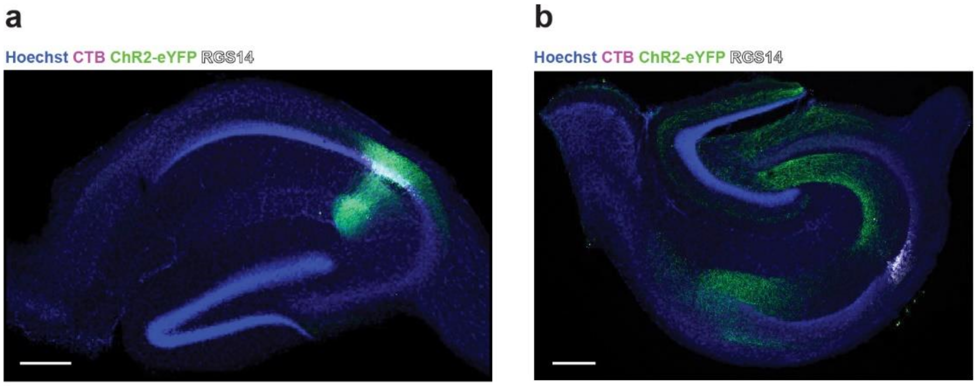

Supplementary Figure 7 | Lack of retrograde CTB labeling in dorsal and ventral HPC contralateral to site of CTB injection in NAc shell.

Images show staining in (a) dorsal and (b) ventral HPC sections contralateral to site of CTB-647 injection in NAc shell. Slices stained for RGS14 (white) and eYFP (labeling fibers from dCA2, green). Note lack of CTB-647 label (magenta) following injections in NAc shell (see Fig. 4e-h for comparison). Scale bars, 250 µm.

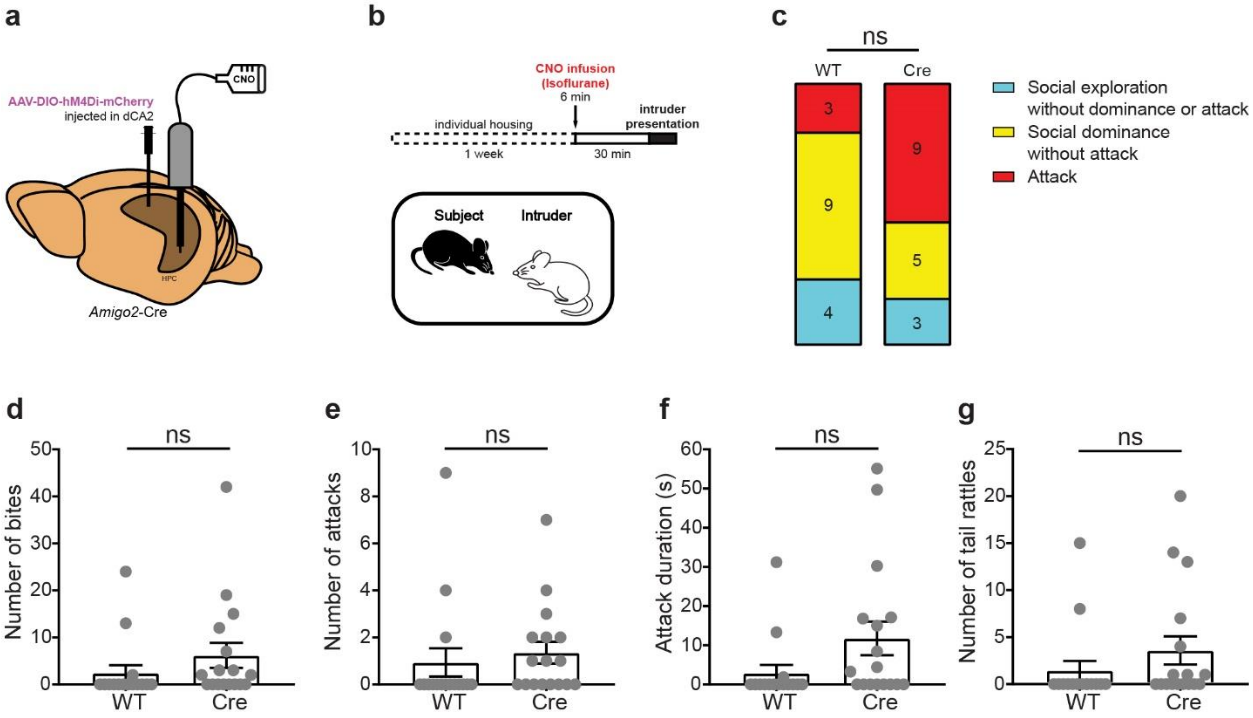

Supplementary Figure 8 | Silencing of dCA2 projections to vHPC does not reduce aggression.

(a) Diagram illustrating viral injection of AAV-DIO-hM4Di-mCherry in dCA2 of *Amigo2*-Cre mice and WT littermates. A cannula guide was implanted in vHPC for CNO infusion. (b) Resident-intruder aggression test. After being individually housed for one week, subject mice were infused with CNO in vHPC and an intruder was placed in the subject mouse home cage. (c) Proportion of mice that engaged in specific behaviors. Numbers of mice indicated in bars. There was no significant decrease in the number of mice engaging in attack with silencing of dCA2 projections (Fisher test: P = 0.1416). No difference was found between groups in: (d) number of bites (unpaired t test: t(13) = 1.178, P = 0.2478); (e) number of attacks (unpaired t test: t(31) = 0.5518, P = 0.5850); (f) attack duration (unpaired t test: t(31) = 1.833, P = 0.0764); or (g) number of tail rattles (unpaired t test: t(31) = 1.166, P = 0.2527). Results in d, e, f and g show mean ± s.e.m. Circles represent individual mice. ns, P>0.05.
